# Oxidation suppression of Cu in alkaline aluminophosphate glass and the effects for radiation-induced luminescence characteristics

**DOI:** 10.1038/s41598-020-78510-z

**Published:** 2020-12-08

**Authors:** Daiki Shiratori, Hirokazu Masai, Takumi Kato, Go Okada, Daisuke Nakauchi, Noriaki Kawaguchi, Takayuki Yanagida

**Affiliations:** 1 Nara Institute of Science and Technology, 8916-5 Takayama, Ikoma, Nara 630-0192 Japan; 2 National Institute of Advanced Industrial Science and Technology, 1-8-31 Midorigaoka, Ikeda, Osaka 563-8577 Japan; 3 Kanazawa Institute of Technology, 3-1 Yatsukaho, Hakusan, Ishikawa 924-0838 Japan

**Keywords:** Lasers, LEDs and light sources, X-rays, Glasses

## Abstract

A glass phosphor is an attractive material for applications in radiation detections because of its high workability and availability with a wide range of chemical compositions. Recently, X-ray-induced luminescence of glasses containing various luminescent activators are actively investigated worldwide. In applications as phosphor, tailoring valence state of activators, which can take multiple valence states in glass, is very important. In this research, we studied effects of glass melting atmosphere on the valence state of copper-activator ion in alkaline aluminophosphate glasses and the radiation-induced luminescence characteristics. Optical absorption and X-ray absorption near edge structure spectra of Cu-doped glasses showed that the glass fused in Ar atmosphere contains higher concentration of Cu^+^ than those prepared in air. In addition, the presence of Cu^+^ enhances the photoluminescence (PL) quantum yield and PL kinetic constant. Furthermore, the increase of Cu^+^ concentration resulted an improvement of the X-ray-induced scintillation and thermally-stimulated luminescence intensity.

## Introduction

Radiation detectors using fluorescent materials have been widely used today, for example, in personal dose monitoring^1–4^, medical diagnosis^5–7^, security^8,9^, high-energy physics^10–12^, environmental dose monitoring^13,14^, and oil logging^15^. Typically, such fluorescent materials are used to convert high energy ionizing radiation into low energy photons^16^ so that the radiation is detected indirectly by reading the converted photons with a photodetector. The material forms of practical radiation detectors are mainly single crystal and ceramic, and glass is seldom used although it has great advantages compared with single crystal and ceramic. For instance, glass has high workability and can be mass-produced easily, and a wide range of chemical compositions are available. Therefore, glass is an attractive candidate for a new fluorescent material to replace existing materials for radiation detection.

Our group has investigated radiation-induced luminescence properties of various phosphate glasses doped with a range of luminescent activators^17–20^. Among these researches, we have focused on Cu^+^ as a luminescence center element since Cu-doped phosphors generally show blue emission due to the 3d^9^4s^1^–3d^10^ transition of Cu^+^, of which the luminescence wavelength agreed well with the spectral sensitivity of common photodetectors. In contrast, it is known that divalent copper does not show emission in the spectral region detectable by conventional photodetectors, and, in fact, Cu ion is likely in a divalent state in a fused glass due to redox reactions. Therefore, to enhance the functionalities as a fluorescent material, it is necessary to prepare the glass with a luminescent activator ion taking the desired valence state (Cu^+^ in case of Cu-doped materials). One of the methods to prevent the formation of Cu^2+^ in glass is to synthesize under a reducing atmosphere during a melting process. In this study, we attempted a suppression of the presence of Cu^2+^ in glass by substituting the molten atmosphere inside a SiO_2_ tammann tube with an inert gas. The glass composition in this study was Li_2_O–K_2_O–Al_2_O_3_–P_2_O_5_ system, where Li_2_O was added to potassium phosphate glass system in order to lower the softening point of the glass because the critical operating temperature of SiO_2_ tammann tubes is about 1100 °C.

The aim of the present study is to investigate the relationship between X-ray-induced luminescence characteristics and the melting atmosphere of Cu-doped glasses. Different melting conditions may cause the emission properties to change based on the redox state of the cation. Based on results of X-ray absorption near edge structure (XANES) analyses of the valence of Cu ions in the glass, correlations between glass melting conditions and optical and X-ray-induced luminescence properties are also discussed.

## Results

The density and thickness of all the specimens are shown in Figure S1. From the XRD measurement results, only a halo peak which is a characteristic of glass was observed, and no crystallization was confirmed (Figure S1). Thermal properties of all the specimens examined by differential thermal analysis (DTA) are shown in Table 1 and Figure S2. Each specimen exhibited an endotherm corresponding to *T*_g_ at around 450 °C, followed by another endotherm corresponding to the re-melting of glass matrix. Thermal annealing was performed for all the glass specimens at the corresponding *T*_g_ temperature to remove the internal stress. The *T*_g_ of the Cu-Ar specimens were higher than those of the Cu-air, suggesting that their thermal properties were different by the melting atmosphere.

**Table 1:** Glass transition temperature *T*_g_ of all the specimens from DTA measurement.

Glass specimens	*T*_g_ (°C)
Cu(0.00%)-Ar	455
Cu(0.01%)-Ar	456
Cu(0.05%)-Ar	455
Cu(0.10%)-Ar	453
Cu(0.00%)-air	457
Cu(0.01%)-air	446
Cu(0.05%)-air	443
Cu(0.10%)-air	442

**Figure 1:**
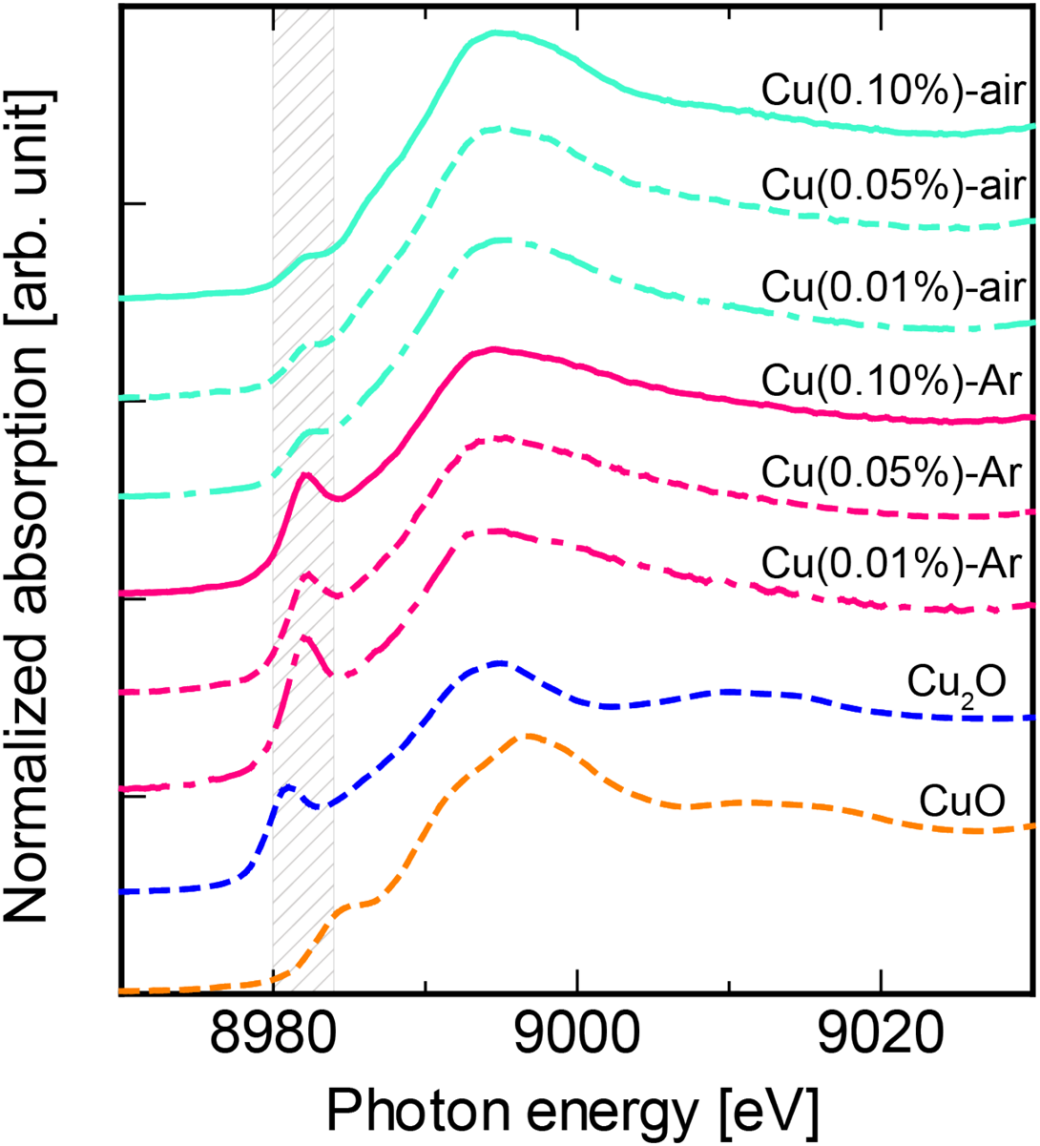
Cu K-edge XANES spectra of the Cu-doped specimens, CuO, and Cu_2_O.

Figure 1 shows normalized Cu K-edge XANES spectra of the Cu-doped specimens. CuO and Cu_2_O are reference raw compounds of divalent and monovalent Cu, respectively, and we estimated the valence state of Cu in the specimens compared with these references. The Cu-Ar specimen and Cu_2_O had a distinct pre-edge around 8981 eV which was assigned to the 1s–4p electronic transition of Cu^21,22^. In contrast, we observed only a shoulder structure in the spectra of the Cu-air specimen and CuO. It has been recognized that the pre-edge feature corresponds to the absorption edge of Cu^+^, but Cu^2+^^23–25^. These results indicate that the Cu-Ar specimen includes most of the Cu in the monovalent state while most Cu ions in the Cu-air specimen was present in the divalent state.

We also estimated the valence state of each Cu-doped specimen quantitatively by the linear combination fitting method using Athena program. In the linear combination fitting method, the X-ray absorption spectrum is approximated by a least-square fitting with a linear combination of spectra of known species. The linear combination fitting has been carried out for the normalized spectrum in the range from 8970 to 9020 eV in this analysis. The fitted spectra and calculated results are shown in the Figure S3 and Table 2. Here, we assumed that all the Cu ions take a state of either CuO (Cu^2+^) or Cu_2_O (Cu^+^), and Cu_total_ is defined as a total number of Cu ions (i.e. sum the numbers of Cu^2+^ and Cu^+^ ions). Comparing between the two different specimens prepared in Ar and air (both are doped with 0.01% Cu), the former includes approximately 90% of Cu ion in the monovalent state while only 20% was found in the latter specimen.

**Table 2:** PL *QY*s, valence ratio, and relative Cu^+^ concentration in the all Cu-doped specimens.

Glass specimens	*QY*s (%)	Valence ratio (Cu^+^/Cu_total_) (%)	Cu^+^ concentration (mol%)
Cu(0.01%)-Ar	19.0	89	0.0089
Cu(0.05%)-Ar	28.3	61	0.0305
Cu(0.10%)-Ar	31.1	67	0.067
Cu(0.01%)-air	5.5	21	0.0021
Cu(0.05%)-air	4.8	11	0.0055
Cu(0.10%)-air	2.3	7	0.007

**Figure 2:**
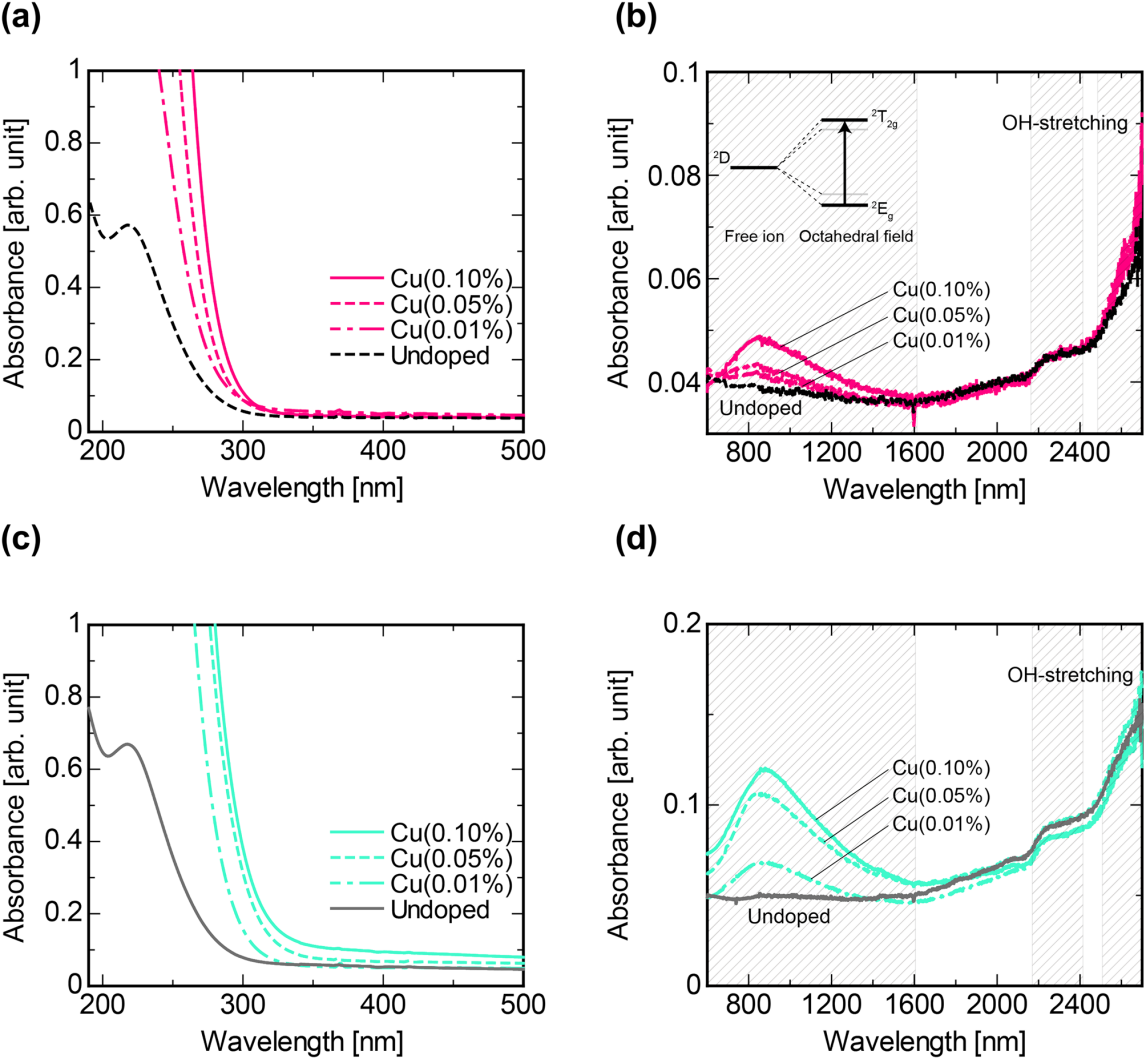
Absorption spectra of all specimens. UV and NIR region absorption spectra of the specimens prepared under (**a**,**b**) Ar and (**c**,**d**) air atmosphere.

Figure 2 shows the optical absorption spectra in the UV and near infrared (NIR) regions for the specimens prepared under (a,b) Ar and (c,d) air. In the undoped specimens, an absorption band feature was observed in the UV region at around 220 nm (Fig. 2a,c). The absorption is originated from unavoidable trace Fe impurities included in the raw starting materials of the glasses^26,27^. For the Cu-doped specimens, an absorption band appeared near 250 nm. The latter absorption band is assigned to 3d^10^–3d^9^4s^1^ transition of Cu^+^^28^. The absorption bands shift to longer wavelength with increasing the Cu concentration. Moreover, the absorption band of the Cu-air specimen appeared on the longer wavelength side than that of the Cu-Ar specimen. This behavior is not attributed to the presence of the 3d^10^–3d^9^4s^1^ transition of Cu^+^ but a charge transfer of O–Cu–O complex^29,30^, which implies that the generation of O–Cu–O is suppressed when the glass is molten in Ar atmosphere. Figure 2b,d are the absorption spectra in the NIR range. The Cu-air specimens showed more remarkable absorption band near 800 nm than those of the Cu-Ar specimens, and any absorption band was not confirmed in the undoped specimens. This absorption band is considered to be due to ^2^E_g_–^2^T_g_ transition of Cu^2+^ placed in an distorted octahedral field^30–32^. This result suggests that Cu^2+^ can be present in a large proportion when the glass was molten in air atmosphere compared with those molten in Ar atmosphere. In other words, the melting under the Ar atmosphere can increase the concentration of Cu^+^. These observations in the absorption spectra as a function of Cu concentration are consistent with those by the linear combination fittings of the XANES spectra. On the other hand, these results also suggest that oxidation does somewhat occurred even when the material was molten in Ar atmosphere. We assume that the oxidation reaction occurred during the quenching process since it was performed in air, although we ensured the glass melt to be exposed to air as short time as possible during the quenching process. An increase in absorbance observed in the region longer than 2500 nm is due to absorption by OH stretching^17^. The absorption attributed to OH stretching was more remarkable in those molten in air than Ar.

**Figure 3:**
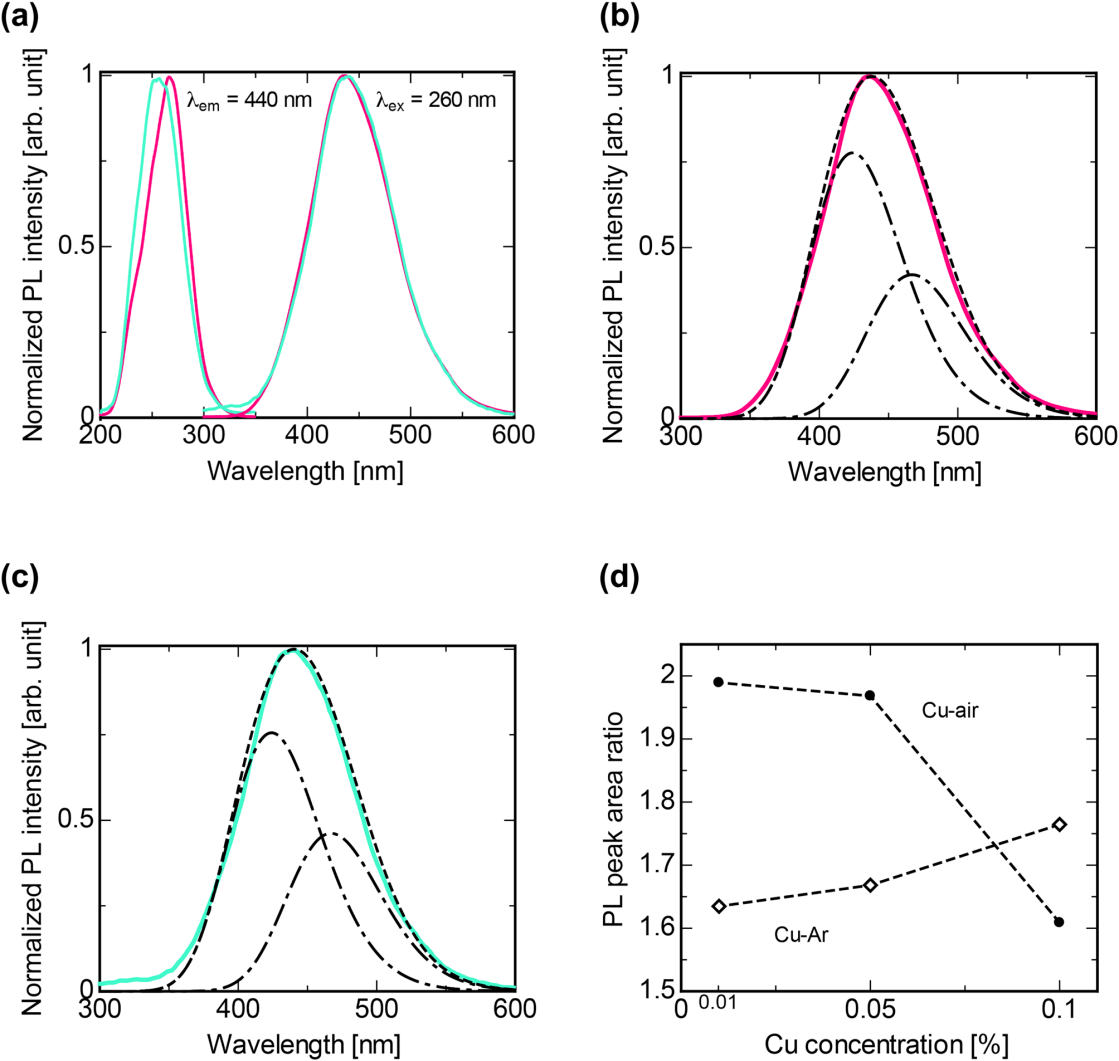
PL excitation/emission spectra of the specimens. (**a**) PL excitation/emission spectra of 0.10% Cu-doped specimens. The monitored and excitation wavelength were 440 nm and 260 nm, respectively. PL spectrum of (**b**) Cu(0.10%)-Ar and (**c**) Cu(0.10%)-air specimens fitted by double gaussians. (**d**) Ratio of the spectral area with a peak at 424 nm to the spectral area with a peak at 467 nm.

Figure 3a shows photoluminescence (PL) excitation/emission spectra of the 0.10% Cu-doped specimens at room temperature (R.T.), and PL excitation/emission contour maps and spectra of all the Cu-doped specimens are also shown in Figure S4 and S5. Each specimen had a broad excitation band in the UV region and showed a broad emission band peaking at around 440 nm. This emission is due to the 3d^9^4s^1^–3d^10^ transition of Cu^+^^31^. Here, we observed a spectral peak shift in the excitation spectra of the Cu-air specimens more effectively than those of the Cu-Ar specimens. The shift in excitation spectra is because the Cu-air specimens have an absorption band due to the O–Cu–O complexes in addition to the 3d^10^–3d^9^4s^1^ band, which overlaps with a part of the excitation band. In contrast, we did not observe a spectral peak shift of an emission band in all the Cu-doped specimens (see Figure S5). In the emission spectra, all the specimens indicated a weak shoulder feature on the longer wavelength side (around 450–500 nm), which suggests that the emission has two different origins. Figure 3b,c show peak deconvolution results by gaussian fitting of 0.10% Cu-Ar and Cu-air. Each spectrum was well-reproduced by a convolution of two gaussians peaking at 424 and 467 nm. Figure 3d is a ratio of the integrated gaussians (424/467 nm) as a function of Cu concentration. The PL band peaking at 424 nm was dominant than the other for all the specimens. In addition, an anticorrelation of the emission ratio was observed between the specimens molten under Ar and air atmospheres. This fact suggests that glass melting atmosphere controls the coordination environment around Cu^+^. The emission origins of these bands will be discussed in detail below together with the results of PL decay constants.

**Figure 4:**
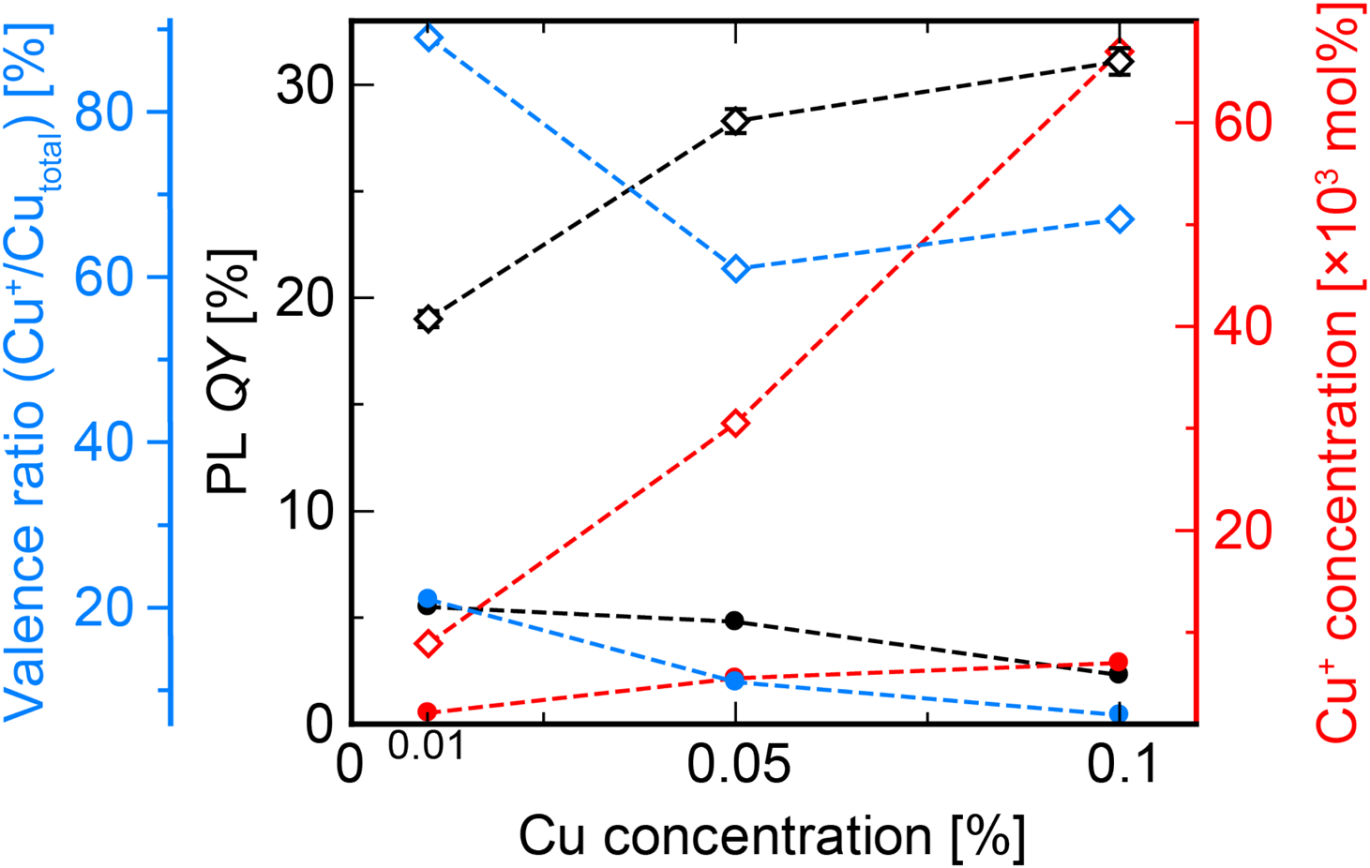
Correlation of PL *QY*, valence ratio (Cu^+^/Cu_total_), and Cu^+^ concentration value as a function of Cu concentration. Open and closed symbols indicate Cu-Ar and Cu-air specimen, respectively.

The maximum PL quantum yields (*QY*) for emission by Cu^+^ are summarized in Table 2. The *QY* of each specimen showed a maximum value when excited at 250 nm. The *QY* was significantly higher in the Cu-Ar specimens than the Cu-air specimens. These results fully reflected the effect of melting in Ar atmosphere. Furthermore, the *QY* of Cu-Ar tended to increase with increasing Cu concentration while it tended to decrease for Cu-air specimens. Figure 4 is a correlation between *QY*, valence ratio (Cu^+^/Cu_total_), and Cu^+^ concentration as a function of doping concertation of Cu. The valence ratio calculated from the linear combination fitting analysis of XANES spectra tends to decrease with increasing the Cu concentration (except for Cu(0.10%)-Ar), while the actual Cu^+^ concentration increases with Cu concentration. Focusing on the relationship between Cu^+^ and *QY*, we can see that there is a positive correlation in the Cu-Ar specimens and a negative correlation in Cu-air.

**Figure 5:**
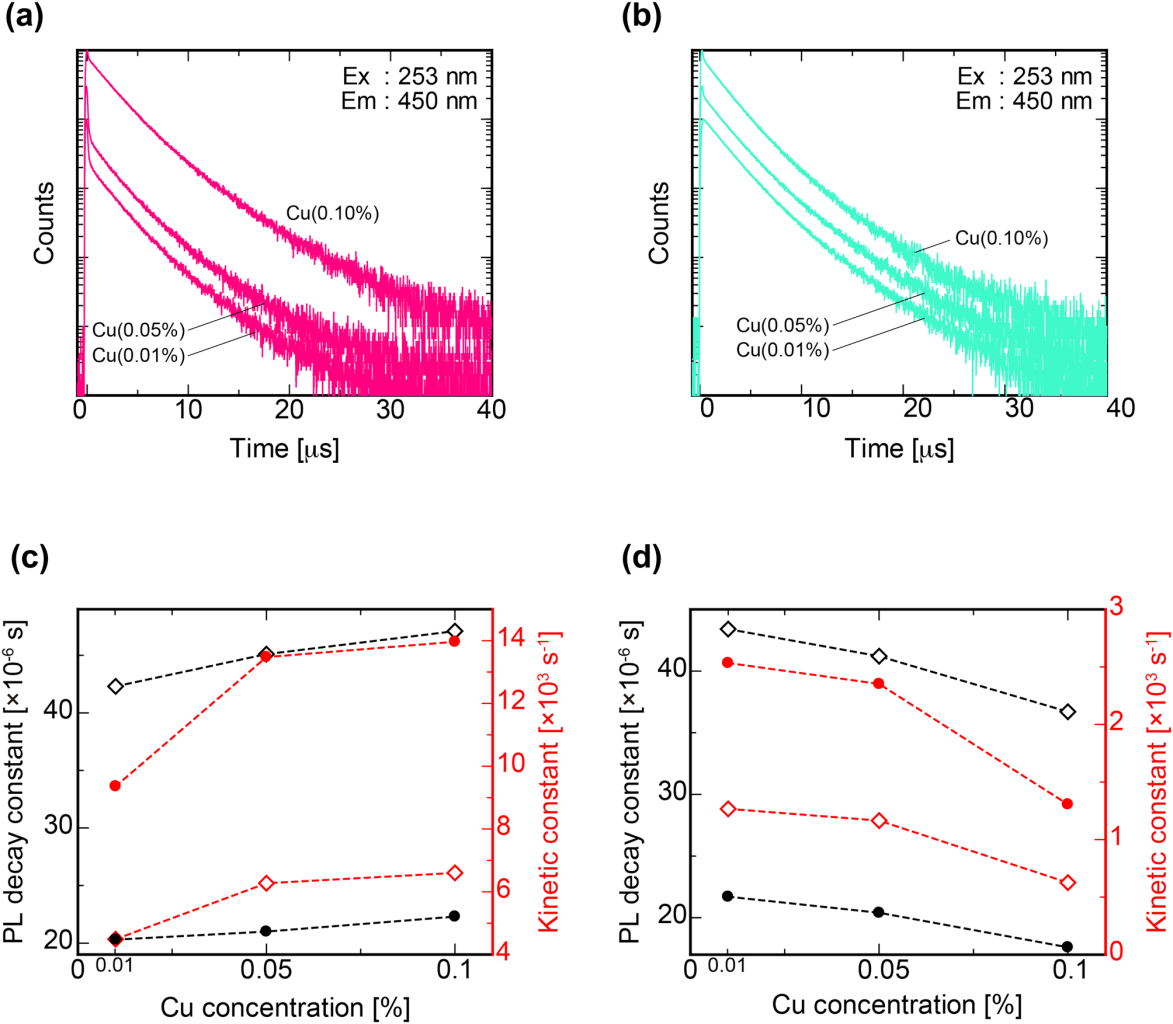
PL decay properties of all the specimens. PL decay curves of the (**a**) Cu-Ar and (**b**) Cu-air specimens. Values of PL decay constants, and kinetic constants as a function of the Cu concentration of the (**c**) Cu-Ar and (**d**) Cu-air specimens. The excitation and monitored wavelength set 260 nm and 440 nm. Open squares and closed circles indicate first and second components of each PL decay constants and kinetic constants, respectively.

Figures 5 represents PL decay features of the (a) Cu-Ar and (b) Cu-air specimens, and the obtained PL lifetime constants are summarized in Table 3. The excitation and monitored wavelengths were 260 and 440 nm, respectively. PL lifetimes were deduced by approximating the experimental data with a sum of two exponential decay functions. The calculated decay constants were on an order of several tens of microseconds, and the experimental results agreed well with the previously reported values of luminescence by Cu^+^ activator^33^. According to a previous study, in case of amorphous materials such as glass, the triplet state (^3^E_g_) of the 3d^9^4s^1^ configuration, which is an excited state of Cu^+^, is split into two levels (T_2g_ and T_1g_) because the Cu coordinates at the distorted tetrahedral sites^33^. The first and second components of the PL lifetime are assigned to the emission transitions from the triplet states T_2g_ and T_1g_ to the ^1^A_1g_ ground state, respectively. The two PL peaks at 424 and 467 nm correspond to the emissions by transitions from T_2g_ and T_1g_, respectively. Figure 5c,d are PL decay constants, and kinetic constants as a function of the Cu concentration of Cu-Ar and Cu-air specimens. Here, the kinetic constant is a probability of radiative transition per unit time and is denoted as *k*_r_ in this paper. Furthermore, if the probability of the non-radiative transition is defined as *k*_nr_, the decay constant can be expressed as *τ* = 1/(*k*_r_ + *k*_nr_). In addition, a PL *QY* is expressed using these kinetic constants as *QY* = *k*_r_/(*k*_r_ + *k*_nr_). From these relationships, the kinetic constant can be calculated by the following equation.

1 $$ \begin{array}{*{20}c} {kr = QY/\tau .} \\ \end{array} $$

**Table 3:** PL decay and kinetic constants of the Cu-doped specimens.

Glass specimens	PL decay constant *τ* (μs)		Kinetic constant *k*_r_ (s^−1^)	
	1st	2nd	1st	2nd
Cu(0.01%)-Ar	20.3	42.3	9.359	4.491
Cu(0.05%)-Ar	21.0	45.1	13.476	6.274
Cu(0.10%)-Ar	22.3	47.1	13.946	6.603
Cu(0.01%)-air	21.7	43.4	2.534	1.267
Cu(0.05%)-air	20.4	41.2	2.352	1.165
Cu(0.10%)-air	17.6	36.7	1.306	0.626

The excitation wavelength was 260 nm which was cut off from light source by using optical filter.

There was a positive correlation between Cu concentration and PL decay constants or kinetic constants of each Cu-Ar specimen. In contrast, we confirmed a negative correlation in Cu-air. This result suggests that the Cu^+^ concentration increases with increasing Cu concentration for Cu-Ar whereas it decreases with increasing Cu concentration in Cu-air.

**Figure 6:**
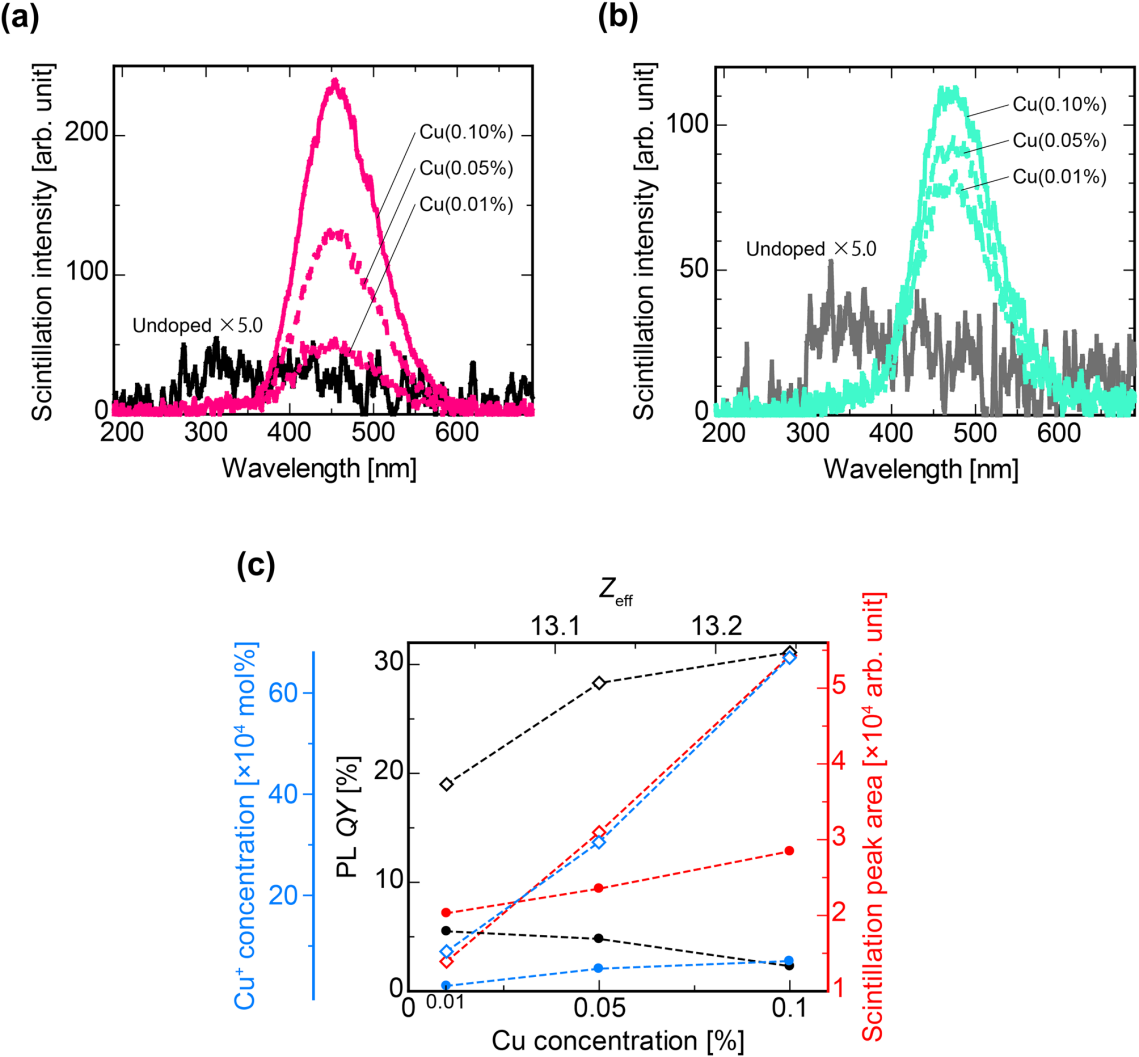
Scintillation properties of all the specimens. X-ray-induced scintillation spectra of the specimens prepared under (**a**) Ar and (**b**) air atmosphere. (**c**) Values of PL *QY*, the Cu^+^ concentration, and the integrated scintillation as a function of *Z*_eff_ and the Cu concentration of the specimens. Open squares and closed circles indicate Cu-Ar and Cu-air specimen, respectively.

Figure 6 shows X-ray-induced scintillation spectra of (a) Cu-Ar and (b) Cu-air specimens, and (c) a relationship between PL *QY*, the Cu^+^ concentration, and the scintillation peak area as a function of effective atomic number (*Z*_eff_: explained in the discussion section) and Cu concentration. In each undoped specimen, we observed a weak broad emission from 300 to 500 nm with a peak around 320 nm. The latter broad emission is attributed to intrinsic luminescence center called L-center in glass^34–36^. In the case of our specimens, this L-center is interpreted as P–O–K linkages^34,37^. In contrast, Cu-doped glass specimens melted both in Ar and air had different emission peak features from the undoped ones. Following the spectral shapes of the PL from Cu-doped specimen, we could attribute the origin of scintillation emission to the 3d^9^4s^1^–3d^10^ transition of Cu^+^. Focusing on the peak position, we can see that these scintillation peak positions are longer than those of PL. Furthermore, the peak wavelength of the Cu-air is longer than that of the Cu-Ar specimens (see Figure S6). Based on a previous report, the reason is probably because an emission peak due to Cu^+^–Cu^+^ dimer appeared in scintillation^38^. The trend of increasing or decreasing scintillation emission area is positively correlated with *QY* among the Cu-Ar specimens. In contrast, it was negatively correlated with *QY* among the Cu-air specimens.

**Figure 7:**
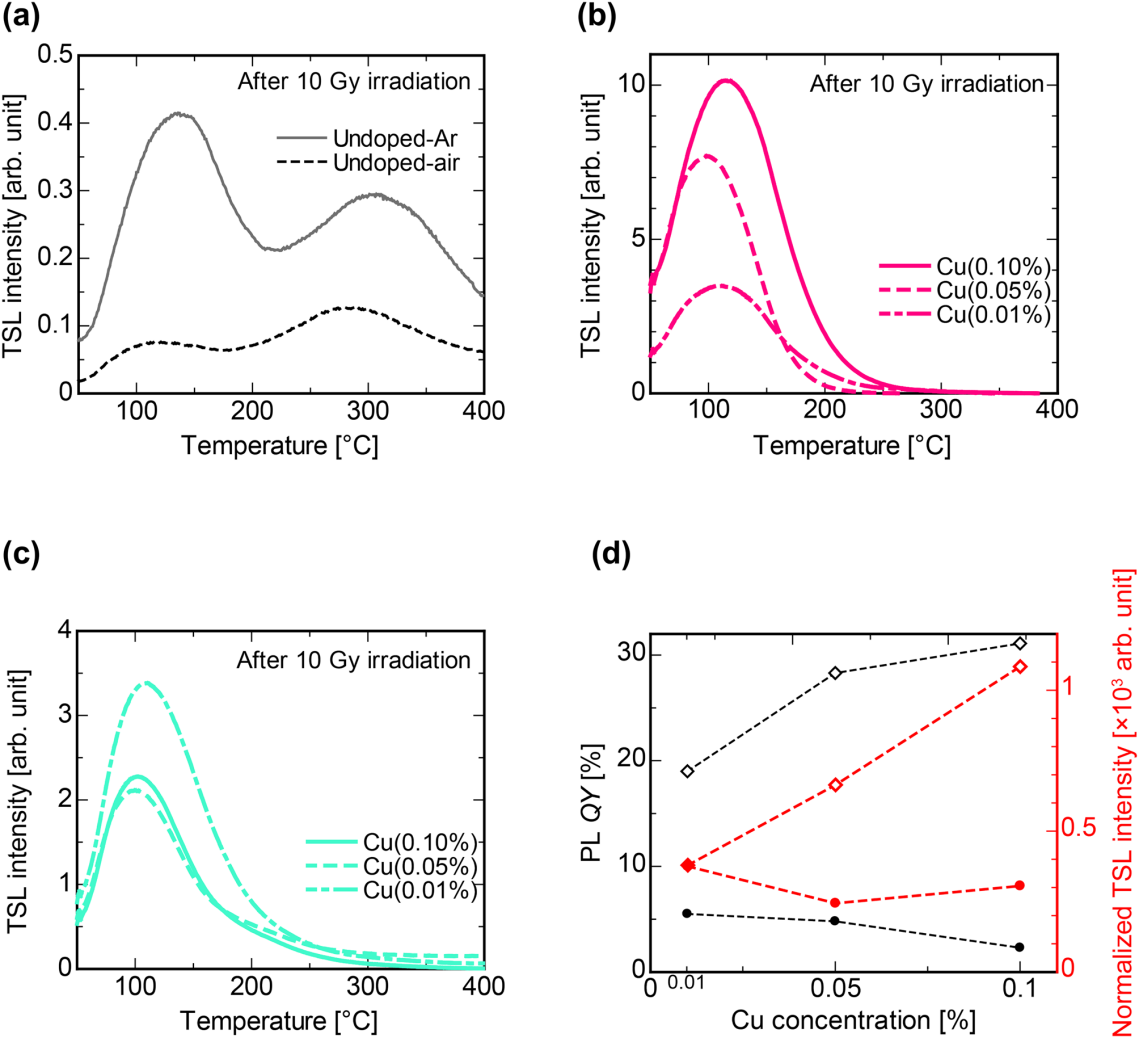
TSL properties of all the specimens. TSL glow curves after 10 Gy X-ray irradiation of the (**a**) undoped, (**b**) Cu-Ar, and (**c**) Cu-air specimens. (**d**) Correlation of PL *QY* in the specimens at each Cu concentration. Open squares and closed circles indicate Cu-Ar and Cu-air specimen, respectively.

Thermally stimulated luminescence (TSL) glow curve represents luminescence intensity as a function of temperature while heating at a constant rate. Prior to the measurement, the specimens were irradiated with X-rays of 10 Gy. The obtained glow curves are illustrated in Fig. 7a–c. We could observe two glow peaks from the undoped specimens. Following the results from scintillation, the luminescence center in TSL of the undoped specimens seems to be the L-center. On the other hand, the Cu-doped specimens showed only a single glow peak, and its intensity significantly increased compared with the undoped specimens. Both Cu-Ar and Cu-air types had a glow peak nearby 110 °C, and no significant differences in the glow curve shape was observed regardless of the difference of the melting atmosphere. It is assumed that the luminescence center in TSL of the Cu-doped specimens is the 3d^9^4s^1^–3d^10^ transition of Cu^+^. Figure 7d shows the PL *QY* and normalized TSL intensity as a function of Cu concentration. In the Cu-Ar specimens, the fluctuation trend of the normalized TSL intensity coincides with *QY*. In the Cu-air specimens, both *QY* and normalized TSL intensity showed a decreasing trend from 0.01 to 0.05% while, from 0.05 to 0.10%, *QY* decreased and normalized TSL intensity increased.

## Discussion

First, we discuss the physical properties of the glass specimens. In the TG–DTA measurements, *T*_g_ of Cu-doped specimens exhibit different tendency depending on the preparation atmosphere. Although *T*_g_ of Cu-doped glass decreases with increasing Cu concentration, the specimens prepared in air atmosphere possess steeper change compared with those prepared in Ar atmosphere. Considering the present results, we assume that Cu^2+^ species are more effective than Cu^+^ to decrease the *T*_g_. We could not confirm metallic Cu related spectrum from the optical absorption and Cu K-edge XANES spectra. Therefore, our glass specimens have not included metallic Cu.

It should be noted that the results of linear combination fitting of the XANES spectra are for reference only because the host material of the fitted spectra is different from that of the reference samples. As illustrated in Fig. 5c,d, PL *QY*s, decay constants and kinetic constants are correlated with Cu concentration. Therefore, actually, the concentration of Cu^+^ in Cu-air is expected to decrease even more strongly with Cu concentration, and the Cu^+^ concentration is expected to show the same decreasing trend as in *QY*. Based on the above observations, we will discuss the results of X-ray-induced luminescence properties.

When X or γ rays pass through a material with atomic number *Z*, the incident radiation decreases exponentially with the penetration depth whose degree is well-defined with a linear attenuation coefficient. The linear attenuation coefficient is proportional to the fourth power of *Z*, since the photoelectric effect is dominant in the case of X-rays with energy of several tens of keV, which is also the case of this research. If the substance is a compound, it is expressed as a sum of the linear attenuation coefficient weighted in weight fractions of each constituent element. In other words, the fourth power of the compound atomic number *Z*_eff_ corresponds to the total sum of products of the weight fraction of each element and the fourth power of respective constituent elements, we can calculate *Z*_eff_ by taking these biquadratic roots. In the present glass composition, an increase in the Cu concentration causes an increase of *Z*_eff_, which in turn increases the effective absorption of X-rays. Therefore, as in Fig. 6c, the scintillation intensity of Cu-air showed an upward trend despite the decrease in the *QY*.

The TSL intensity of the Cu(0.10%)-Ar specimen was greater than that of the Cu(0.10%)-air specimen. Here, the luminescence efficiency *η*_i_ in TSL is defined by the following equation^39^:

2 $$ \begin{array}{*{20}c} {\eta_{{\text{i}}} = n_{{{\text{eh}}}} \;\eta_{{{\text{tr}}}}\; \eta_{{{\text{TSL}}}}\; h\nu \eta_{{{\text{esc}}}} .} \\ \end{array} $$

Here $n_{{{\text{eh}}}}$, $\eta_{{{\text{tr}}}}$, $\eta_{{{\text{TSL}}}}$, $h\nu$, and $\eta_{{{\text{esc}}}}$ are defined as the number of e–h pairs per unit mass and per unit absorbed dose, the fraction that is captured in traps which can be thermally-stimulated, TSL efficiency, the average energy of the emitted TSL photons, and the fraction of the produced photons that will escape without being absorbed, respectively. In addition, $\eta_{{{\text{TSL}}}}$ is also defined as a product of a release probability of the charges from the traps: *p*, a transport efficiency of released charges to a luminescent center: *S*, and a recombination efficiency on the luminescent center (i.e. PL *QY*). Now, since eacspecimen has the same base material, $n_{{{\text{eh}}}}$, *p*, *S*, and $h\nu$, are consistent whereas $\eta_{{\text{i}}}$ depends only on $\eta_{{{\text{tr}}}}$, *QY*, and $\eta_{{{\text{esc}}}}$. Therefore, the TSL intensity of the Cu(0.10%)-Ar specimen with high *QY* and low self-absorption by Cu^2+^ was higher than that of the Cu(0.10%)-air specimens. Figure 7d shows that the TSL intensity increased with increasing Cu concentration in the Cu-Ar specimen while the TLS intensity decreased from 0.01% to 0.05% in the Cu-air specimen and again increased from 0.05% to 0.10%. Considering the formula for $\eta_{{\text{i}}}$, it is reasonable to think that the increase of TSL intensity depends on $\eta_{{{\text{tr}}}}$. because the integrated TSL intensity is not being correlated with *QY*. In the Cu-air specimens, the Cu^+^ concentration did not increase with increasing Cu concentration of the specimens, however, the concentration of Cu^2+^ increased. This suggests that Cu^2+^ is likely to function as an electron trap center. As a result, the TSL intensity is predominantly contributed by the *QY* at and below 0.05% while the number of electron trapping centers (i.e. Cu^2+^) dominantly contributes at and above 0.05% of Cu concentration.

## Conclusion

The absorption spectra of the glass molten under the air atmosphere showed a pronounced optical absorption attributed to the ^2^E_g_–^2^T_2g_ transition of Cu^2+^ whereas we could scarcely observe the absorption band in the glass fabricated by melting in Ar atmosphere. Furthermore, as a result of analyzing XANES at the copper K-edge, we found that most of the copper in the glass have a low valence state (Cu^+^) when they are molten in the Ar atmosphere. Fabrication of a glass under a reducing atmosphere improved the PL *QY* of Cu^+^ luminescence. Along with an increase of luminescence efficiency, TSL intensity of the Cu-doped specimen improved. These results showed that the control of the melting atmosphere is effective for improving radiation-induced luminescence characteristics due to luminescent center ion such as copper which can assume several valence states.

## Methods

### Preparation of undoped and Cu-doped lithium potassium alminophosphate glass

Li_2_O–K_2_O–Al_2_O_3_–P_2_O_5_ glasses were prepared by the conventional melt-quenching method using an electric furnace. The starting materials of Li_3_PO_4_ (2N), (KPO_3_)_n_ (2N), and Al(PO_3_)_3_ (4N) were uniformly mixed at the molar ratio of 10:30:60, respectively. Thus, the obtained chemical composition of glass was 8.8Li_2_O–8.8K_2_O–17.7Al_2_O_3_–64.7P_2_O_5_ (mol%). To investigate dopant concentration dependence on luminescence properties of the glass specimens, Cu_2_O (2N) was added to the host material to include 0, 0.01, 0.05, and 0.10 mol.% of Cu ion while the host material is dealt as 100%. In this paper, ‘Cu concentration’ indicates the nominal value. These powders were weighed in a total amount of 10.0 g and mixed homogeneously using a mortar. The mixed powder was put into a Pt crucible and then melted at 1100 °C for 20 min in Ar or air atmosphere. The glass preparation under an Ar gas atmosphere was carried out by the following procedure. The mixed powder in a Pt crucible was loaded into an electric tube furnace in which a SiO_2_ tammann tube is equipped. The tammann tube was vacuumed to less than − 0.1 MPa, and then a high purity Ar gas (5N) was purged. The above vacuum and purge processes were repeated three times to ensure all the air was removed. During the glass melting process, a flow of Ar gas was controlled at a rate of 0.5 L/min. After the melting for 20 min, the electric furnace was opened in air, and the Pt crucible was quickly taken out from the furnace using a tongue to quench the glass melt on a stainless-steel plate preheated to 200 °C. After cooling to R.T., the glasses were annealed at the glass transition temperature *T*_g_ for 1 h in air. Following the annealing procedure, the glass specimens were cut into the dimension of 9.0 × 9.0 ×  ~ 1.4 mm^3^ and then the surfaces were mechanically polished. In this paper, we denote that the obtained glasses molten in Ar are Cu-Ar specimens while those molten in air are Cu-air specimens. The *T*_g_ of the glass specimen was determined using a TG–DTA system (Rigaku) operating at a heating rate of 10 °C/min. The densities of the glass samples were determined by the Archimedes method using analytical balances (GR-120, A&D Company, Limited). XRD patterns were measured by MiniFlex 600 (Rigaku).

### Cu K-edge XANES measurement

In order to evaluate valence states of Cu in the glass specimens prepared under Ar and air atmosphere, we have measured XANES at the BL01B1 beamline of the synchrotron radiation facility, SPring-8. The Cu K-edge XANES measurements were carried out using a Si (111) double-crystal monochromator in the fluorescence mode using 19-SSD at R.T. Reference samples of CuO and Cu_2_O, which were prepared by mixing the granular sample with boron nitride, were also measured in the transmittance mode.

### Optical/luminescence properties

Transmittance spectra of the glass specimens were measured using a spectrophotometer (V670, JASCO) across a spectral range from 190 to 2700 nm with 1 nm intervals. The PL excitation/emission spectra were measured by a spectrofluorometer (FP-8600, JASCO) which equipped a Xe-lamp. The range of monitored wavelength was from 300 to 600 nm while the excitation wavelength was 260 nm. Excitation spectra was measured from 200 to 350 nm when the monitored wavelength was 440 nm. PL *QY*s were measured using Quantaurus-QY (C11347, Hamamatsu Photonics) at R.T. *QY* includes the measurement errors in ± 2%. The monitored excitation/emission wavelength ranges were 250–350 and 350–750 nm with 10 nm intervals, respectively. To determine an origin of luminescence, PL lifetime measurements were performed using Quantaurus-Tau (C11367, Hamamatsu Photonics).

### X-ray-induced scintillation measurement

X-ray-induced scintillation spectra were measured using our laboratory-made setup^40^. We used an X-ray generator (XRB80N100/CB, Spellman) and CCD-based detector (Shamrock 163 monochromator and DU-420-BU2 CCD, Andor).

### TSL measurement

TSL glow curves were measured using TL-2000 (Nanogray Inc.), and we set up temperature range and heating rate to 50–400 °C and 1 °C/s, respectively^41^.

## Supplementary information

Supplementary Informations.
