# Supplementary material for: Oxidation suppression of Cu in alkaline aluminophosphate glass and the effects for radiation-induced luminescence characteristics
Source: Sci Rep. 2020 Dec 8;10:21403. doi: 10.1038/s41598-020-78510-z (PMC7722858; doi:10.1038/s41598-020-78510-z)
Supplement: Supplementary file 1 — Supplementary Informations. [file 41598_2020_78510_MOESM1_ESM.docx]

**Oxidation suppression of Cu in alkaline aluminophosphate glass by melting under Ar atmosphere and its effects for radiation-induced luminescence characteristics**

D. Shiratori, H. Masai, T. Kato, G. Okada, D. Nakauchi, N. Kawaguchi, and T. Yanagida

**Supplementary Data**

| **Supplementary Table 1 Density and thickness of the all specimens.** Density and thickness include the measurement errors in ±0.002% and ±0.015%, respectively. | | |
| --- | --- | --- |
| Glass specimen | Density [g/cm^3^] | Thickness [mm] |
| Undoped: Ar | 2.51 | 1.528 |
| 0.01% Cu: Ar | 2.51 | 1.692 |
| 0.05% Cu: Ar | 2.52 | 1.386 |
| 0.10% Cu: Ar | 2.52 | 1.384 |
| Undoped: air | 2.51 | 1.453 |
| 0.01% Cu: air | 2.51 | 1.312 |
| 0.05% Cu: air | 2.53 | 1.347 |
| 0.10% Cu: air | 2.54 | 1.264 |

| 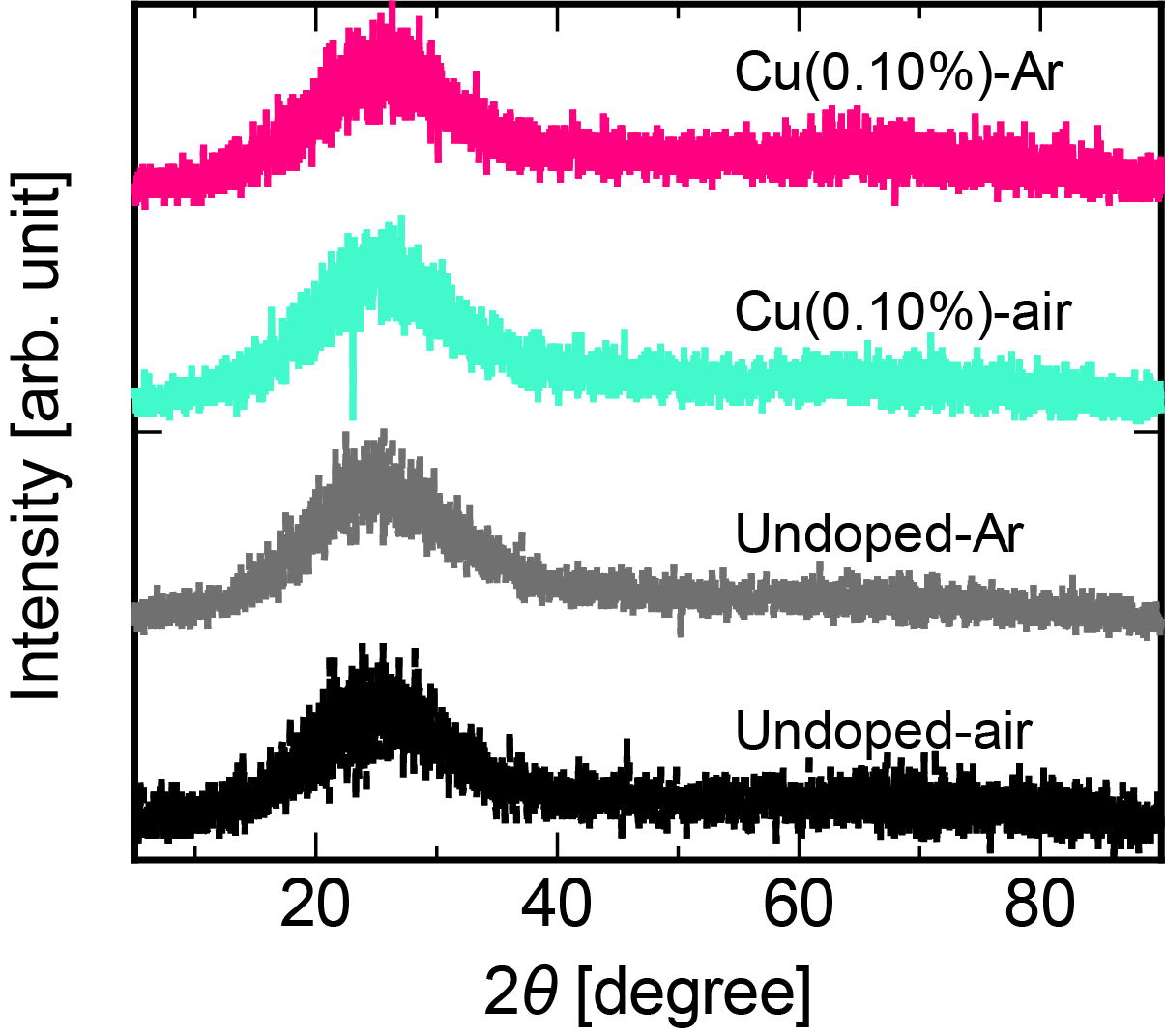 |
| --- |
| **Supplementary Figure 1 \| X-ray diffraction (XRD) patterns of the 0.1% Cu-doped and undoped specimens.** |

| 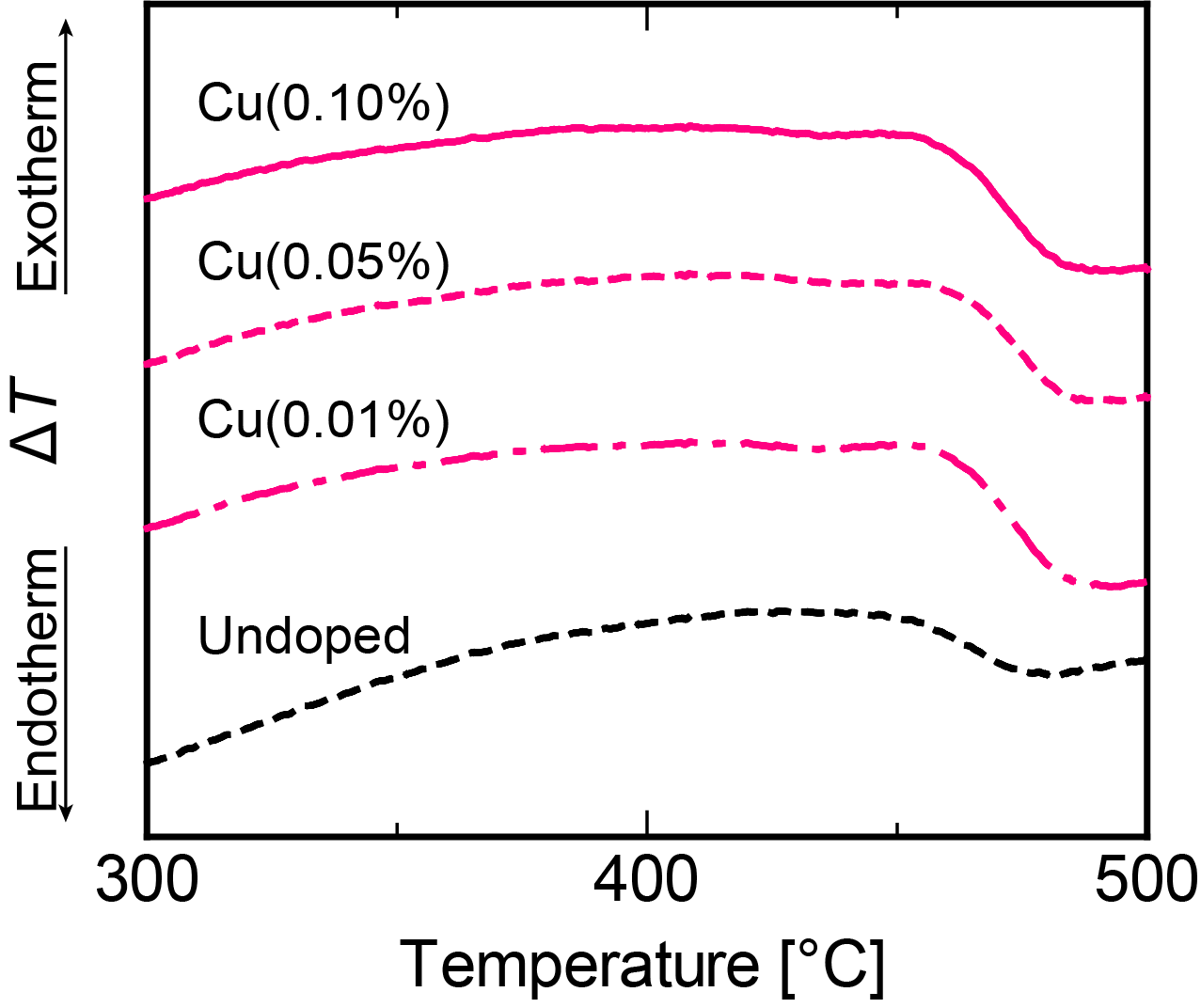  **(a)** |
| --- |
| 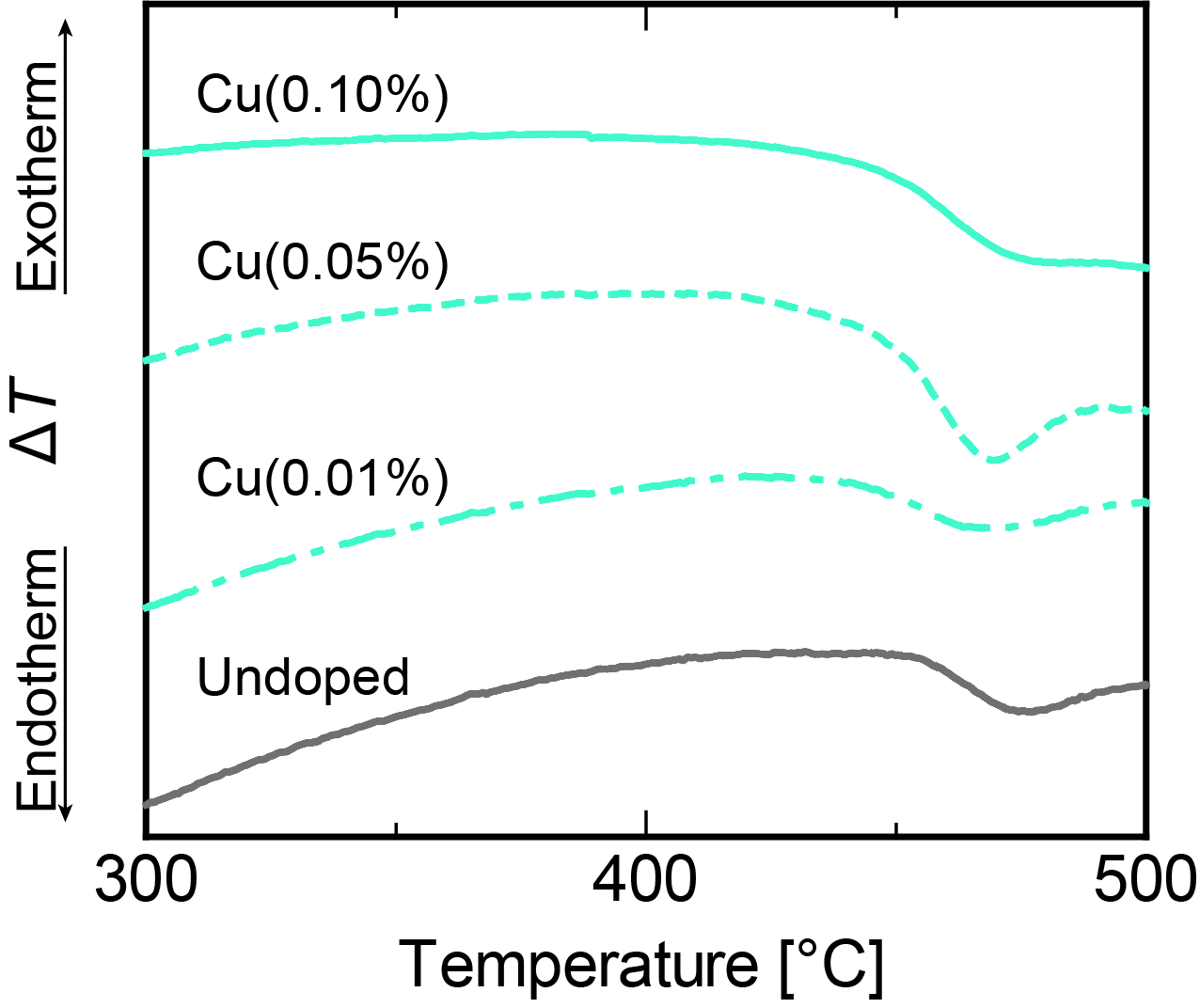  **(b)** |
| **Supplementary Figure 2 \| differential thermal analysis (DTA) curves for the specimens prepared under (a) Ar and (b) air atmosphere.** |

| 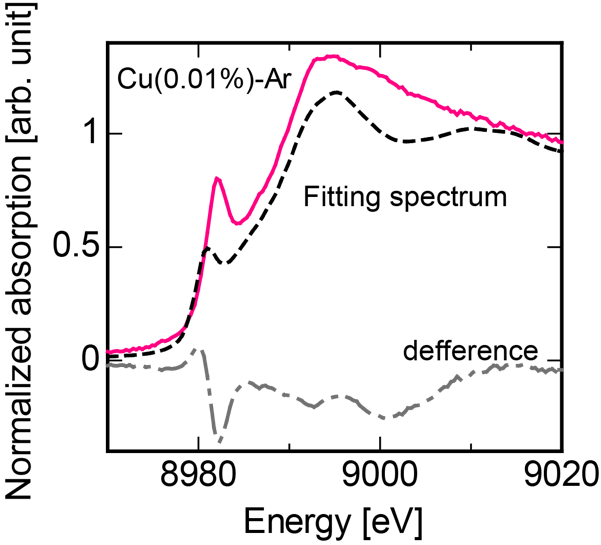  **(a)** | 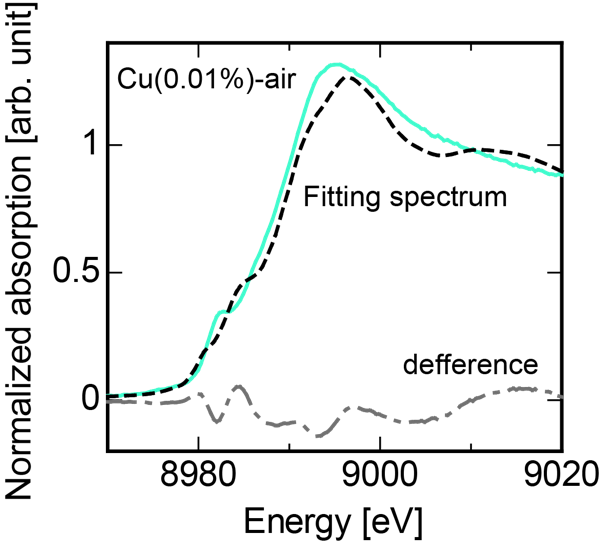  **(b)** |
| --- | --- |
| 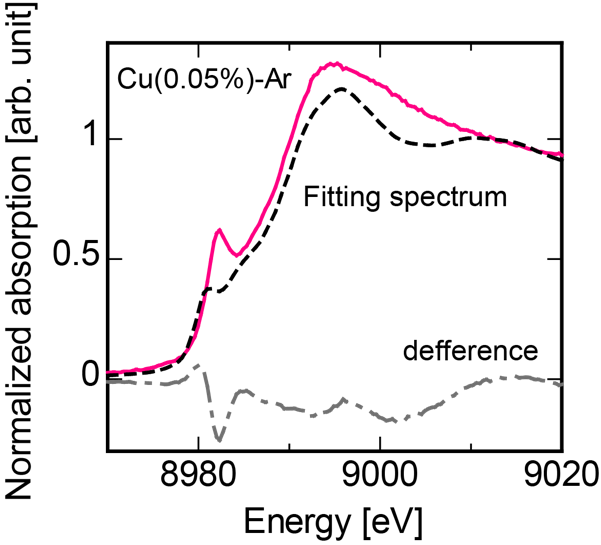  **(c)** | 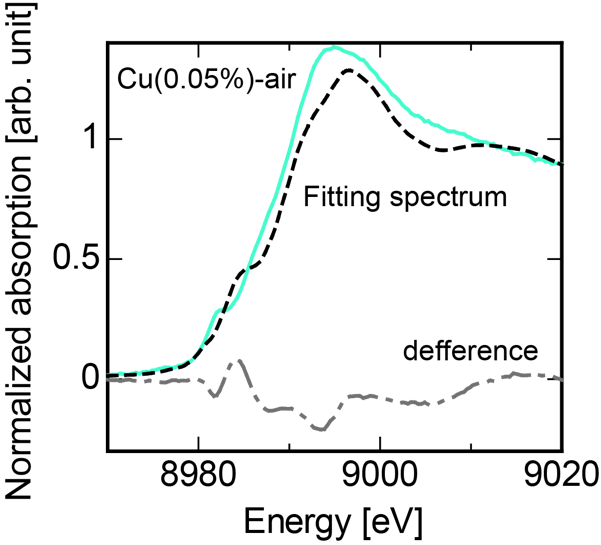  **(d)** |
| 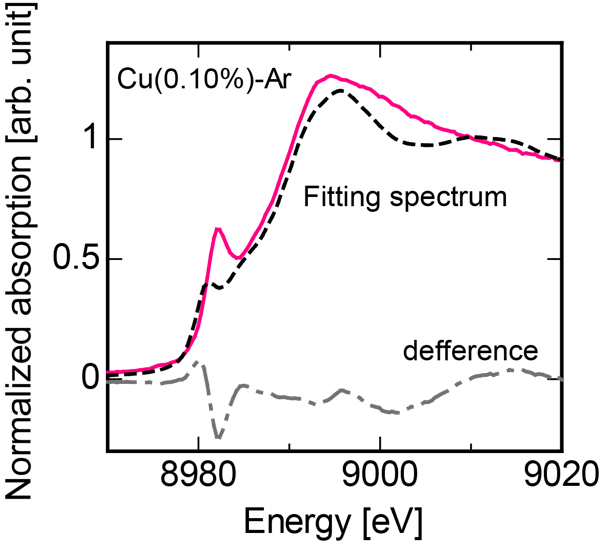  **(e)** | 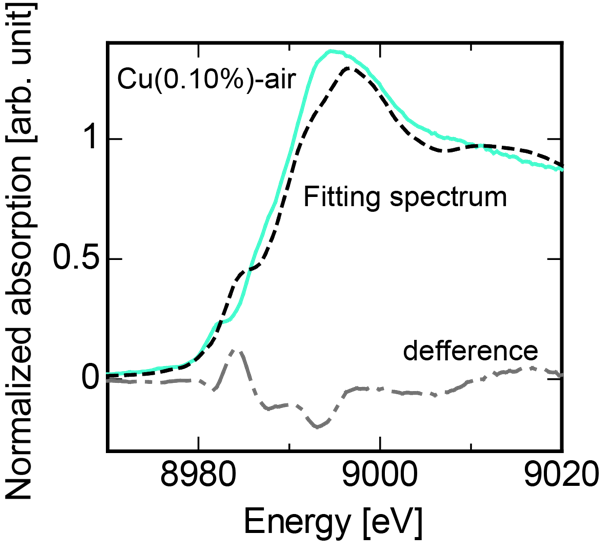  **(f)** |
| **Supplementary Figure 3 \| Cu K-edge X-ray absorption near edge structure (XANES) spectra and linear combination fitting results of all Cu-doped specimens.** The correspondence with each of the samples in (a) to (f) is as follows: (a) Cu(0.01%)-Ar, (b) Cu(0.01%)-air, (c) Cu(0.05%)-Ar, (d) Cu(0.05%)-air, (e) Cu(0.10%)-Ar, (f) Cu(0.10%)-air. Solid lines show the spectra of each specimens, dashed lines show the proximate spectra by the linear combination fitting, and single-dotted lines show the fitting errors. | |

| 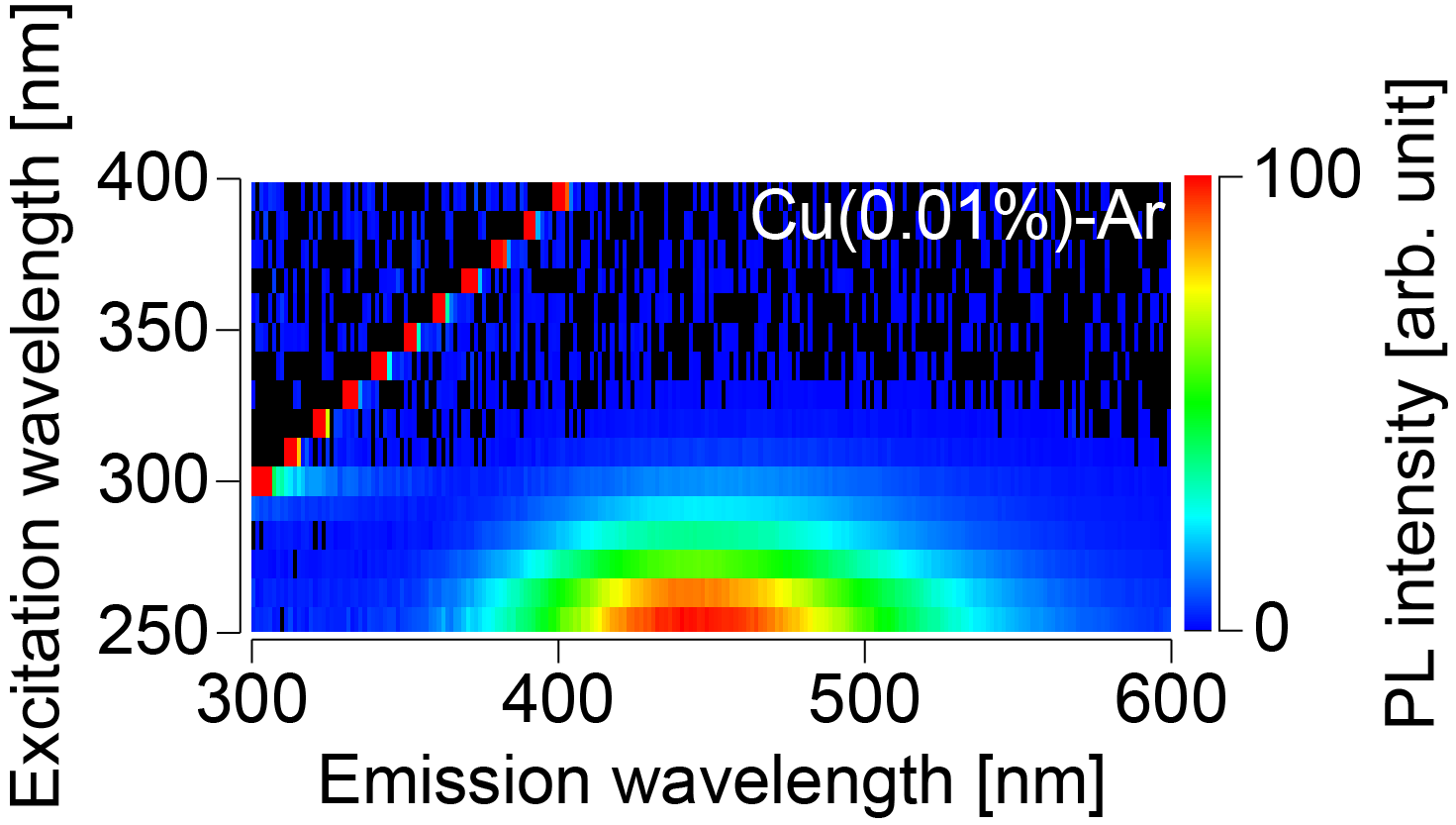  **(a)** | 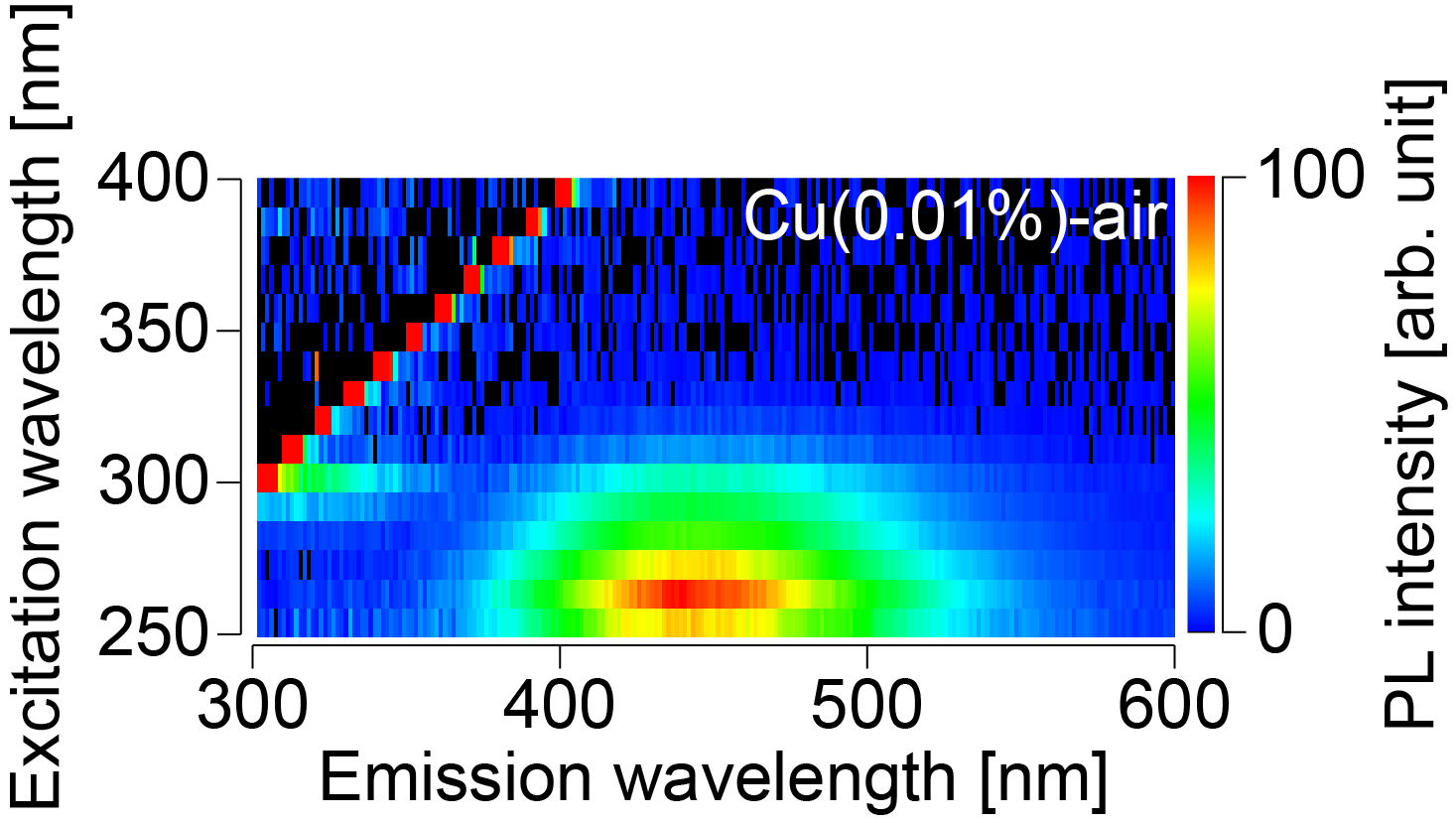  **(b)** |
| --- | --- |
| 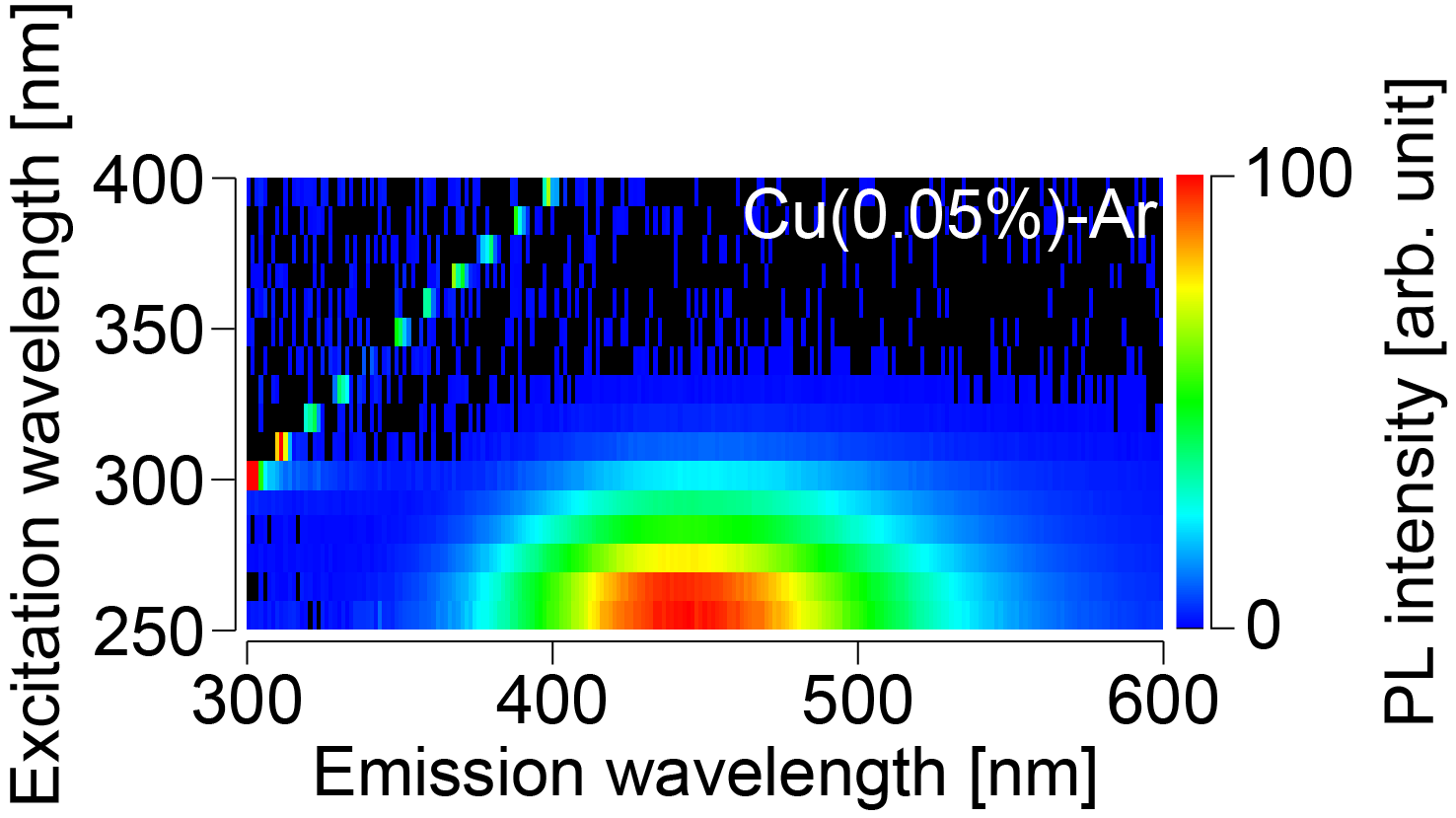  **(c)** | 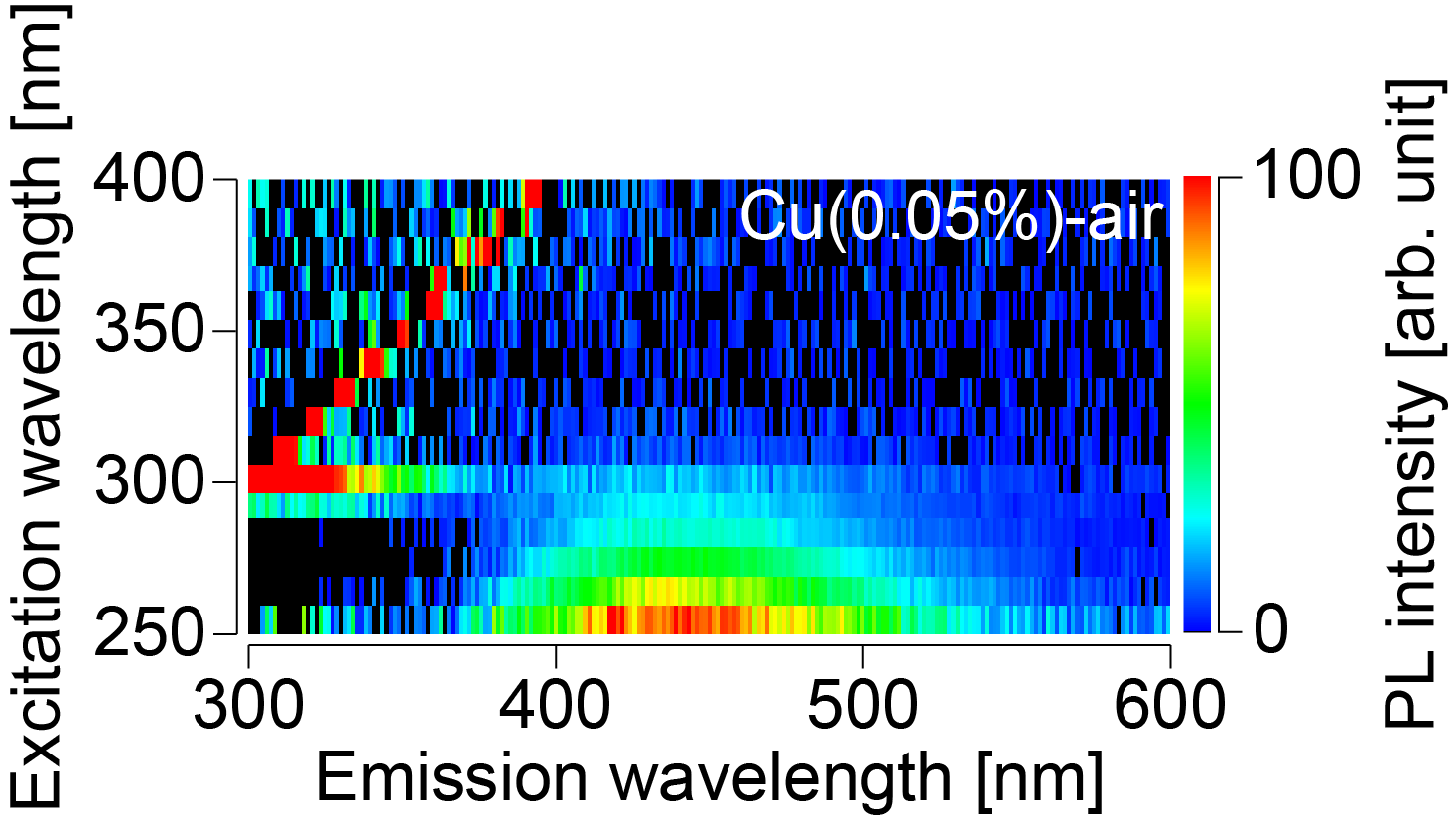  **(d)** |
| 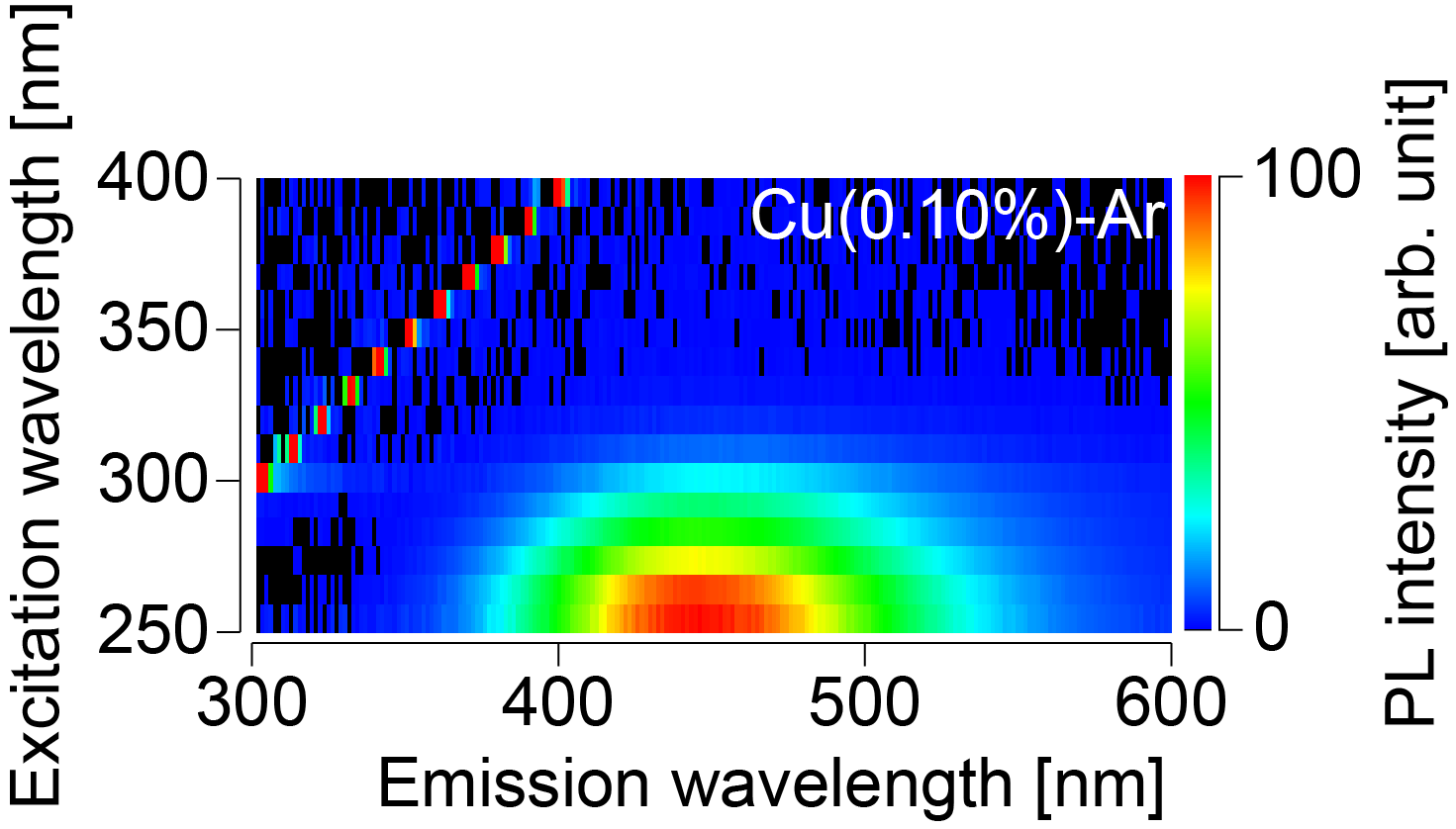  **(e)** | 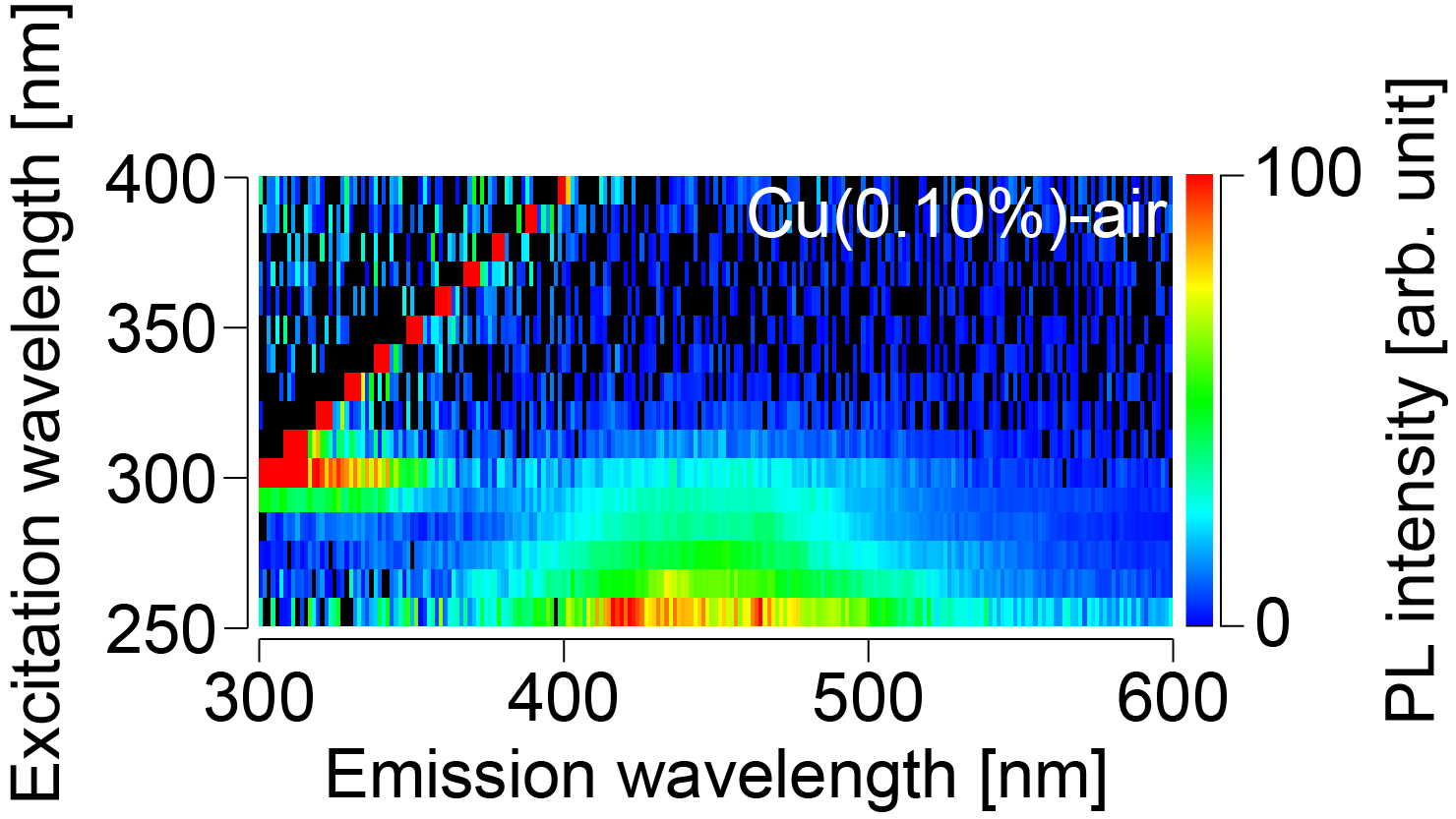  **(f)** |
| **Supplementary Figure 4 \| Photoluminescence (PL) excitation/emission contour maps of all Cu-doped specimens.** The correspondence with each of the samples in (a) to (f) is as follows: (a) Cu(0.01%)-Ar, (b) Cu(0.01%)-air, (c) Cu(0.05%)-Ar, (d) Cu(0.05%)-air, (e) Cu(0.10%)-Ar, (f) Cu(0.10%)-air. The vertical and horizontal axes indicate the excitation and emission wavelengths, respectively. | |

| 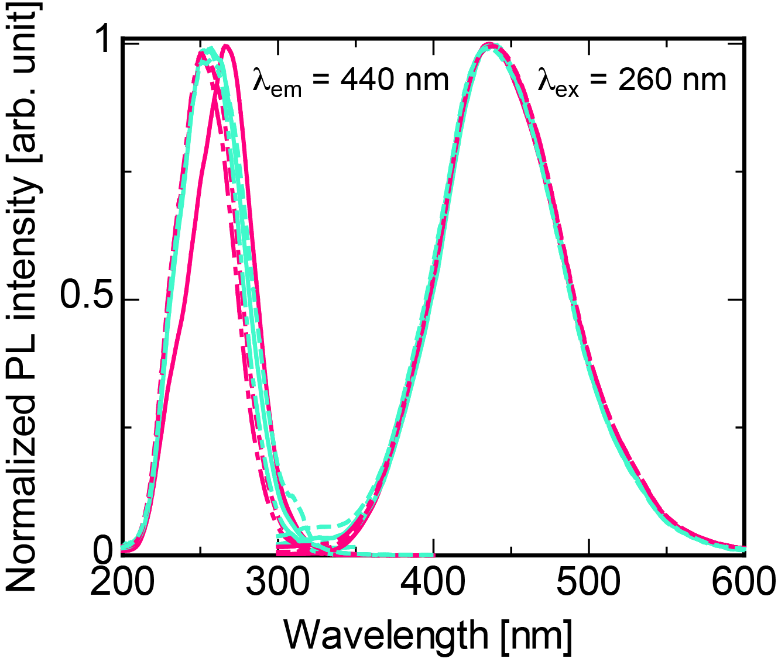 |
| --- |
| **Supplementary Figure 5 \| PL excitation/emission spectra of all the Cu-doped specimens.** The pink and green spectra show the specimens molten under Ar and air atmosphere, and solid, dashed and single-dotted lines show the spectra of the 0.10%, 0.05% and 0.01% Cu-doped samples, respectively. The observed wavelength of the excitation spectra was 440 nm, and the excitation wavelength of the emission spectra was 260 nm. |

| 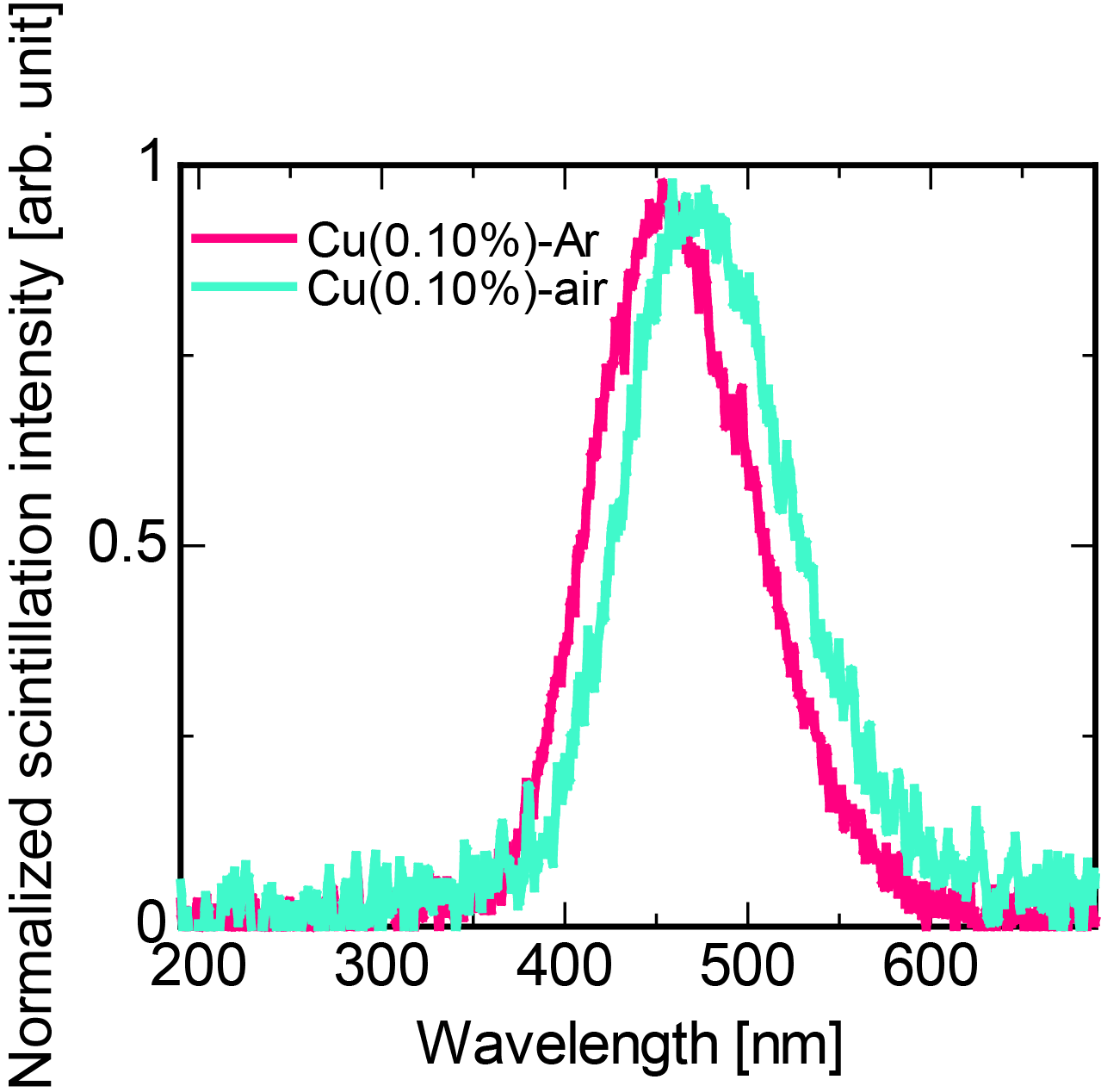 |
| --- |
| **Supplementary Figure 6 \| X-ray-induced scintillation spectra of the 0.10% Cu-doped specimens.** |
